# Pathways to schizophrenia: Divergent effects of childhood trauma and polygenic risk on cognition and subcortical dynamics

**DOI:** 10.1192/j.eurpsy.2026.12246

**Published:** 2026-07-29

**Authors:** Danqing Huang, Yicheng Long, Zhening Liu, Wenjian Tan, Xiawei Liu, Yunzhi Pan, Feiwen Wang, Lena Palaniyappan

**Affiliations:** 1 Department of Psychiatry, and National Clinical Research Center for Mental Disorders, The Second Xiangya Hospital, Central South University, China; 2 Department of Psychiatry, McGill University, Montreal, Canada; 3 Douglas Mental Health University Institute, Department of Psychiatry, McGill University, Montreal, Canada

**Keywords:** childhood trauma, dynamic functional connectivity, polygenic risk scores, resting-state fMRI, exposome

## Abstract

**Background:**

Schizophrenia is often a persistent mental illness characterized by cognitive deficits and altered brain network dynamics especially affecting the cortico-subcortical salience processing system. Childhood trauma has been implicated in mechanisms contributing to schizophrenia, but the role of genetic vulnerability remains unclear.

**Methods:**

A total of 164 schizophrenia patients and 114 healthy controls were included. First, a matched subsample of 117 patients (76 with and 41 without childhood trauma), and 59 healthy controls were compared on polygenic risk scores for schizophrenia (SczPRS). SczPRS was then correlated with neurocognitive performance using a comprehensive cognitive battery (*n* = 147 patients) and brain-wide static and dynamic functional connectivity (sFC and dFC) using resting-state fMRI (*n* = 117 patients). Patients were also stratified into high-SczPRS and low-SczPRS (*n* = 25 each) subgroups.

**Results:**

Patients with childhood trauma had significantly lower SczPRS than those without. Higher Childhood Trauma Questionnaire (CTQ) scores related to lower cognitive PC score (*r* = −0.282, *p* < 0.001), but CTQ did not affect brain-wide dysconnectivity or subcortical/salience system among patients. Patients with the highest SczPRS showed lower dFC between the cingulo-opercular network and subcortical networks (*F* = 7.949, *p* = 0.007), but SczPRS did not affect cognitive performance.

**Conclusions:**

Our findings reveal distinct effects of childhood trauma and polygenic risk in the neural and cognitive substrates of schizophrenia. Childhood trauma relates to poor cognitive function, while polygenic risk primarily affects salience/subcortical network dynamics. These patterns indicate that in genetically predisposed individuals who experience childhood trauma, a “double mechanistic hit” may influence the schizophrenia phenotype.

## Introduction

Dopamine hypothesis [1–5] has been the most influential of all prevailing neurobiological theories of schizophrenia. At a network level, the dysconnectivity involving subcortical structures such as striatum and thalamus on the one hand and fronto-insular structures constituting the salience network and cingulo-opercular network (CON) on the other is linked to the dopamine-mediated salience processing dysfunction seen in schizophrenia [6–10]. Based on these observations, Harikumar linked this network dysconnectivity pattern to neurotransmitter and stress/vulnerability models rather than the neurodevelopmental aspects of psychosis [11].

Childhood trauma is emerging as a major risk factor for psychosis in recent studies [12]. It is associated with up to a threefold increase in the lifetime risk of developing a psychotic disorder, with the risk escalating with the number and severity of the exposure [13–15] and influencing cognitive performance [16], symptom severity [17], and treatment outcomes [18]. Some accounts describe a distinct neurodevelopmental effect of this trauma, affecting overall cognitive functions that may in turn predispose to psychosis [19, 20].

Most individuals who experience childhood trauma do not develop psychosis. This suggests that genetic vulnerability to psychosis may play an important role. Polygenic risk score (PRS) [21], a widely used genetic measure of susceptibility, aggregates alleles associated with an illness from many loci into an independent score. SczPRS explains 8–12% [22] of the additive genetic liability for schizophrenia. Interestingly, genetic predisposition to schizophrenia may also increase the risk of exposure to childhood trauma in ways that are poorly understood yet. Guloksuz [23] found an additive interaction between PRS and some (emotional abuse/neglect, sexual abuse) but not the other aspects of childhood trauma in the risk of schizophrenia [24]. Recent evidence indicates that the genetic susceptibility to trauma may overlap with the SczPRS, even if SczPRS per se may not be high among those with trauma [25]. It is not clear if the two risk factors (SczPRS and childhood trauma) converge on common mechanisms (e.g., salience related subcortical–cortical connectivity) or have an add-on effect due to two distinct mechanisms, that is, one affecting general cognitive development and the other affecting the salience system.

Using resting-state fMRI, studies have related both trauma [26–30] and SczPRS [31] to functional dysconnectivity in schizophrenia. Most of these findings are based on the assumption that functional connectivity (FC) remains stable across the period of scanning. However, FC fluctuates over time [32, 33] and these fluctuations [34, 35], termed dynamic functional connectivity (dFC), provide valuable insights into the brain’s functional organization by capturing its subtle temporal dynamics [32, 36–38] that are not detectable via static FC analysis. Aberrant dFC has been reported in schizophrenia [39–41], especially affecting the salience processing cingulo-opercular system [42, 43].

**Figure 1.**
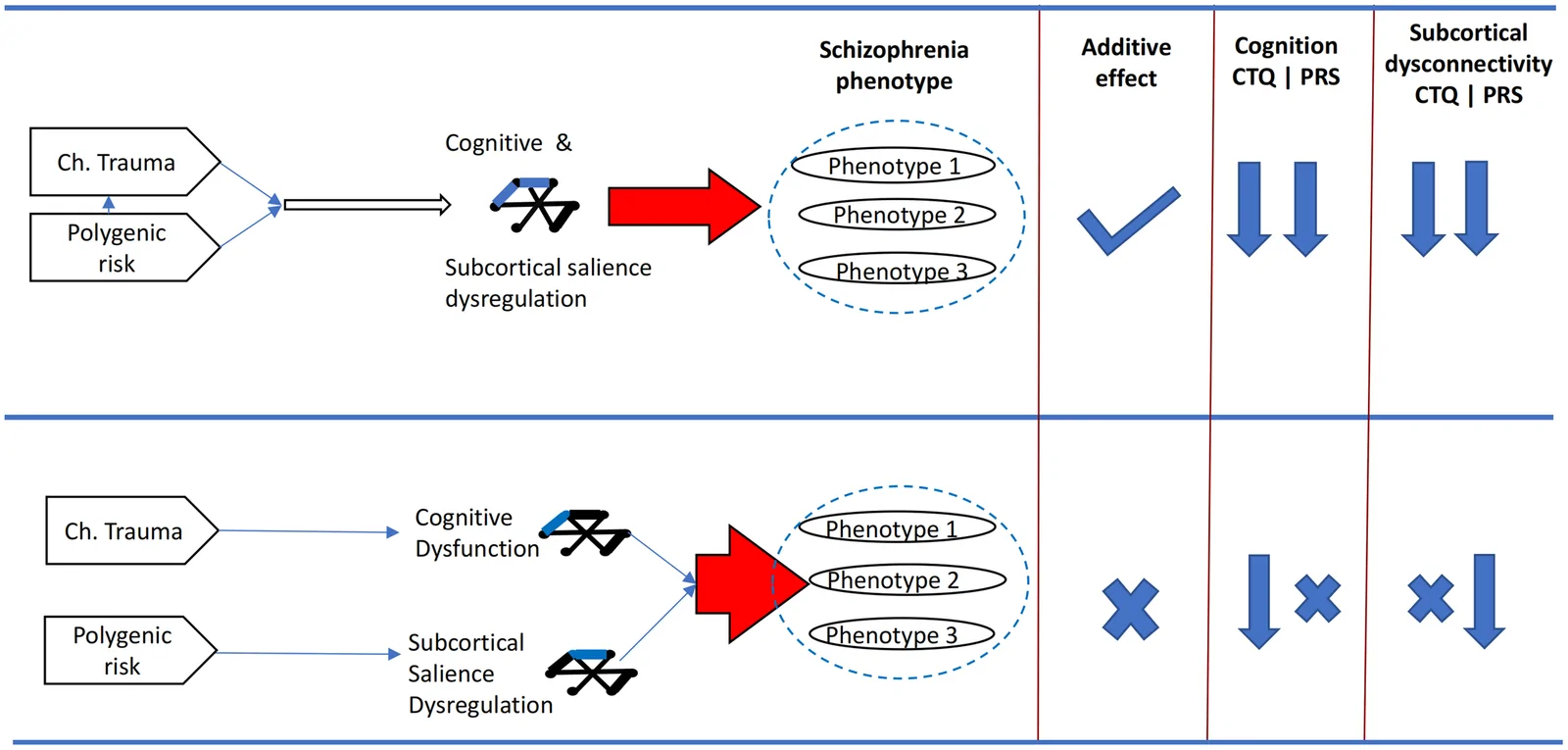
Two mechanistic routes of the effect of childhood trauma on schizophrenia. We tested if both trauma (CTQ) and polygenic risk (PRS) of schizophrenia converged on salience dysregulation indexed by subcortical dysconnectivity and cognitive dysfunction (top panel), or if they had separable effects on these two pathways (bottom panel). In the presence of convergence, we expected trauma to relate to higher PRS and schizophrenia polygenic risk to subcortical dysconnectivity, and in the absence of convergence, we expected trauma to relate primarily to cognition and schizophrenia polygenic risk to subcortical dysconnectivity. *Note*: CTQ, childhood trauma questionnaire; PRS, polygenic risk score for schizophrenia. Figure 1. long description.

To better understand how childhood trauma and genetic factors contribute to the neurobiological mechanisms of schizophrenia, it is important to reduce confounds due to normally prevalent risk factors, such as cannabis use, that can affect the same mechanistic pathways [44–46] and the effect of illness chronicity, which can result in a convergence of initially heterogenous pathways [47, 48]. The present study examined the associations among childhood trauma, PRS, brain functional connectivity, and cognitive function in patients with schizophrenia <30 years of age, without cannabis exposure. Specifically, we test three associations: (1) individuals with higher PRS may be more likely to report experiences of childhood trauma (converging effect); (2) both childhood trauma and PRS may converge on cognitive impairments, and (3) both contribute to alterations in cortico-subcortical functional connectivity that is relevant to dopaminergic signaling. Observations refuting these may support divergent effects whereby the two risk factors operate on distinct mechanisms but result in overlapping phenotype (Figure 1). On extrapolation, individuals with both higher PRS and trauma may only have a “single hit” on their risk of schizophrenia if the effects are convergent but a “double hit” if they are not. To test these hypotheses, we estimated SczPRS and childhood trauma exposure and employed static functional connectivity (sFC) and dFC analyses alongside cognitive assessments. Our results indicate that genetic vulnerability and childhood trauma may influence distinct pathways toward the risk of schizophrenia.

## Materials and methods

### Study participants

One hundred and sixty-four patients with schizophrenia, recruited from the Second Xiangya Hospital of Central South University, were involved in this study. And 114 healthy controls were recruited from the community, with a modest reimbursement provided for their time and travel expenses. The sample size was based on the available cohort with complete clinical, genetic, cognitive, and neuroimaging data. All participants were of Chinese ancestry. Written informed consent was obtained from all participants. For patients with schizophrenia, the study procedures were explained to both the patients and their legal guardians, and written consent was obtained from the patients and/or their legal guardians as appropriate. The study was approved by the Ethics Committee of the Second Xiangya Hospital, Central South University (Approval No. (2020) Ethics Review [Research] No. 036). All procedures complied strictly with the Declaration of Helsinki. Only right-handed patients aged less than 30 years meeting DSM-5 criteria for schizophrenia were included. Patients with other schizophrenia-spectrum or psychotic disorders were not included. We excluded people with (1) a history of additional mental disorders (e.g., PTSD or pervasive developmental disorders); (2) substance abuse, dependency, or a history of severe physical illness (except nicotine use); (3) metallic implants, including electronic devices or any other MRI contraindications; and (4) a history of neurological conditions. The criteria for healthy individuals were identical to those for the schizophrenia group, with no controls satisfying the DSM-5 criteria for any mental disorders.

### Clinical assessment

Trained psychiatrists evaluated the severity of clinical symptoms in schizophrenia using the Positive and Negative Syndrome Scale (PANSS) [49]. The PANSS, consisting of 30 items, includes three subscales: negative symptoms, positive symptoms, and general psychopathology, which evaluate the severity of symptoms over the past month. Each item is scored on a 7-point scale, with 1 point representing no symptoms and 7 points indicating the most severe symptoms, providing a comprehensive assessment of the patient’s condition.

### Cognitive function assessment

For the cognitive function assessment, participants completed a comprehensive evaluation using a battery of tests designed to assess memory, attention, processing speed, executive function, and problem-solving abilities. The assessment included several subtests from the Wechsler Adult Intelligence Scale (WAIS) [50], the Wechsler Memory Scale-Revised (WMS-R) [51], other tasks designed to assess specific cognitive domains, and the Modified Wisconsin Card Sorting Test (MWCST) [52]. Further details are provided in the Supplementary Material S1.

To evaluate overall cognitive performance, principal component analysis (PCA) was applied to the cognitive measures. Missing values were imputed using the mean within each group (schizophrenia with childhood trauma, schizophrenia without childhood trauma, and healthy controls). The first principal component (PC1), explaining the largest proportion of variance, was extracted as a composite score representing global cognitive function.

### Assessment of childhood trauma

Participants who consented were evaluated for childhood trauma exposure using the Chinese short-form Childhood Trauma Questionnaire (CTQ) [53], a validated [54] and widely used [28, 55] self-report measure. The internal consistency of the CTQ in the schizophrenia sample was Cronbach’s *α* = 0.770. The CTQ comprises 25 clinical items, organized into five subscales, with each subscale containing five items that assess different forms of childhood trauma: emotional abuse, emotional neglect, physical abuse, physical neglect, and sexual abuse. Responses are rated on a 5-point Likert scale, from 1 (never) to 5 (always), reflecting the frequency of the reported events. Following previous studies [28, 56–58], participants were classified as having experienced childhood trauma if their scores exceeded the cutoff for any subscale: emotional abuse ≥13, emotional neglect ≥15, physical abuse ≥10, physical neglect ≥10, and sexual abuse ≥8. Using this classification, schizophrenia patients were divided into two subgroups: those with childhood trauma and those without. For analyses involving the three groups classified by CTQ (schizophrenia with childhood trauma, schizophrenia without childhood trauma, and healthy controls), a total of 76 schizophrenia patients with childhood trauma, 41 schizophrenia patients without childhood trauma, and 59 healthy controls were included. There were no significant differences among the three groups in terms of age, sex, education, or head motion.

### Imaging data acquisition and preprocessing

For each participant, the resting state functional MRI and T1-weighted structural imaging data were acquired using a 3.0 T Siemens scanner. Further details are provided in the Supplementary Material S2.

Data preprocessing was carried out using the DPARSF software [59, 60] (http://rfmri.org/DPARSF) following a standard pipeline. The steps included removing the first 10 volumes, applying slice-timing correction, performing motion realignment, spatial normalization, and temporal filtering (0.01–0.10 Hz) while also regressing out covariates, such as cerebrospinal fluid and white matter. After preprocessing, trained researchers manually checked the image quality. To ensure data quality, the mean framewise displacement (FD) was calculated for each participant, and all of the images underwent manual inspection to confirm the absence of significant artifacts or distortions. Participants with excessive head motion (mean FD > 0.2 mm) or poor-quality images (identified through manual checking) were excluded from the current study.

### Edge-based FC of static and dynamic brain network

In this study, the preprocessed fMRI images were parcellated into 264 nodes throughout the brain based on the 264 regions of interest (ROIs) specified in the well-validated and widely utilized Power functional atlas [61–64]. Between brain nodes, the edge-based FC was defined by the *z*-transformed Pearson correlation coefficients, calculated from the average time series of each pair of nodes [65, 66]. According to previous studies [67–69], all of the nodes were grouped into 10 distinct networks including the auditory network (AUD), CON, default-mode network (DMN), dorsal attention network (DAN), frontoparietal network (FPN), salience network (SAN), sensorimotor network (SMN), subcortical network (SUB), ventral attention network (VAN) and visual network (VN).

The dynamic functional brain networks were constructed using the Power functional atlas following the same procedure as the static brain network analysis, after preprocessing the data. For each of the 264 nodes, rs-fMRI signals from all of the voxels within the defined ROIs were extracted and divided into multiple time windows using a sliding-window method. Based on previous recommendations [28, 39, 70], a fixed window length of 100 s with a 6-s step was employed. Within each time window, functional connectivity (FC) between pairs of nodes was calculated using Pearson correlation coefficients, which were then transformed into Fisher’s *z*-scores, yielding a 264 × 264 symmetric connectivity matrix for each time window [71, 72]. To assess temporal variability, the variance of the FC strength for each edge was computed across all time windows, reflecting the degree of fluctuation over time. A higher variance indicated greater temporal variability in the dynamic FC of the respective edge [73, 74].

### Targeted gene sequencing and PRS calculation

The target gene selection, sequencing, and PRS calculation followed the methodology that has been most used to date and described in previous studies on Chinese participants [75]. The target genes comprised variants located within 1 mb upstream and downstream of 108 schizophrenia risk loci (128 genes) identified by the latest genome-wide association study (GWAS) conducted by the Schizophrenia Working Group of the Psychiatric Genomics Consortium [76]. Both coding and regulatory noncoding regions were targeted for sequencing, with the regulatory regions encompassing the 3′ un-translated region (3′UTR), 5′ un-translated region (5′UTR) and intron-exon boundaries (25 bp).

Genomic DNA was extracted from 2 mL of ethylene diamine tetra-acetic acid (EDTA) anticoagulated peripheral blood, fragmented and used for sample library construction (Illumina Hiseq) according to the manufacturer’s instructions. The Hardy–Weinberg equilibrium (HWE) was assessed in the control group for each marker using Pearson’s χ^2^ test (1 degree of freedom). Schizophrenia-associated single nucleotide polymorphisms (SNPs) were identified via χ^2^ tests using PLINK [77], with Fisher’s exact test applied throughout the study to mitigate distributional bias. The PRS for the target regions was constructed using the PRSice toolbox [78] based on GWAS analysis.

### Statistics

#### Analyses using the matched sample

One-way analysis of variance (ANOVA) was used to compare age, years of education, and head movement (mean FD) across the three groups: schizophrenia with childhood trauma, schizophrenia without childhood trauma, and healthy controls. χ^2^ tests were conducted to examine sex distribution differences among the groups. Additionally, *t*-tests were performed to compare PANSS scores between the two schizophrenia groups (with and without childhood trauma).

The differences in PRS among the three groups were analyzed using ANOVA, followed by least significant difference (LSD) post-hoc tests. Meanwhile, to quantify the effect size of the PRS difference between the two schizophrenia groups, Cohen’s *d* was calculated.

To compare edge-based sFC and dFC differences among the three groups, analysis of covariance (ANCOVA) was performed with sex, age, education, and head motion as covariates. The results were considered significant at *p* < 0.05 after false discovery rate (FDR) correction.

To compare the edge-based sFC and dFC values in subcortico-cortical networks (SUB-DMN, SUB-FPN, SUB-CON, SUB-SAN, SUB-VAN, and SUB-DAN) between the two schizophrenia groups (with and without childhood trauma), ANCOVA was conducted with age, sex, education, and mean FD as covariates. FDR correction was applied for multiple comparisons, with significance set at *p* < 0.05. Additionally, ANCOVA was performed to compare the same six networks between the top and bottom 25 schizophrenia patients based on PRS, using the same covariates and significance threshold.

#### Analyses using the whole sample

In the entire sample of schizophrenia patients, we first examined the correlation between the cognitive principal component score (cognitive PC score) and the CTQ total score. We then examined the correlations between the five CTQ subscale scores and the cognitive PC score, with FDR correction applied within this set of tests. Additionally, we analyzed the correlations between CTQ scores and each individual cognitive measure, again applying FDR correction within this set of tests. Corrected *p* < 0.05 was considered statistically significant. Furthermore, the correlation between the cognitive PC score and PRS was also calculated.

Exploratory PRS × CTQ interaction analyses were performed using linear regression for the cognitive PC score and the six subcortico-cortical dFC outcomes. PRS and CTQ scores were standardized before computing the interaction term. The cognitive model included the main effects of PRS and CTQ, the PRS × CTQ interaction term, age, sex, and education, whereas the dFC models additionally included mean FD as a covariate.

As a supplementary sensitivity check for the extreme-group PRS comparison, continuous PRS was tested in relation to CON-SUB dFC using ANCOVA, with age, sex, education, and mean FD included as covariates.

Partial correlation analyses were conducted to examine associations between CON-SUB dFC and PANSS total, positive, negative, and general psychopathology scores, controlling for age, sex, education, and mean FD.

## Results

### Participant characteristics

Details of the demographic and clinical characteristics of participants were shown in Table 1. A total of 76 schizophrenia patients with childhood trauma, 41 schizophrenia patients without childhood trauma, and 59 healthy controls were included in the analyses.

**Table 1.** The demographic, CTQ, and clinical characteristics of three groups Table 1. long description.

	SZ with childhood trauma (*n* = 76), mean ± SD	SZ without childhood trauma (*n* = 41), mean ± SD	HC (*n* = 59), mean ± SD	Group comparisons
Age (years)	18.63 ± 2.59	18.65 ± 3.04	19.01 ± 1.94	*F* (2, 173) = 0.441, *p* = 0.644
Education (years)	11.51 ± 2.23	11.67 ± 1.95	12.14 ± 2.78	*F* (2, 173) = 1.180, *p* = 0.310
Sex (male/female)	40/36	20/21	34/25	χ2 (2) = 0.793, *p* = 0.673
Mean FD (mm)	0.074 ± 0.034	0.077 ± 0.038	0.066 ± 0.027	*F* (2, 173) = 1.685, *p* = 0.188
CTQ scores	58.37 ± 10.45	44.05 ± 4.41	48.27 ± 8.28	*F* (2, 173) = 43.252, *p* < 0.001
PANSS scores	67.99 ± 22.16	61.98 ± 19.17	/	*t* (115) = 1.465, *p* = 0.146

Abbreviations: CTQ, childhood trauma questionnaire; HC, healthy control; mean FD, mean framewise displacement; PANSS, positive and negative syndrome scale; SD, standard deviation; SZ, schizophrenia.

No significant differences were found in age, sex, years of education, or head motion among three groups (all *p* > 0.05). There was no significant difference in PANSS scores between the two schizophrenia groups. The details of cognitive function are presented in Supplementary Table S1.

### Additive burden of PRS and CTQ

**Figure 2.**
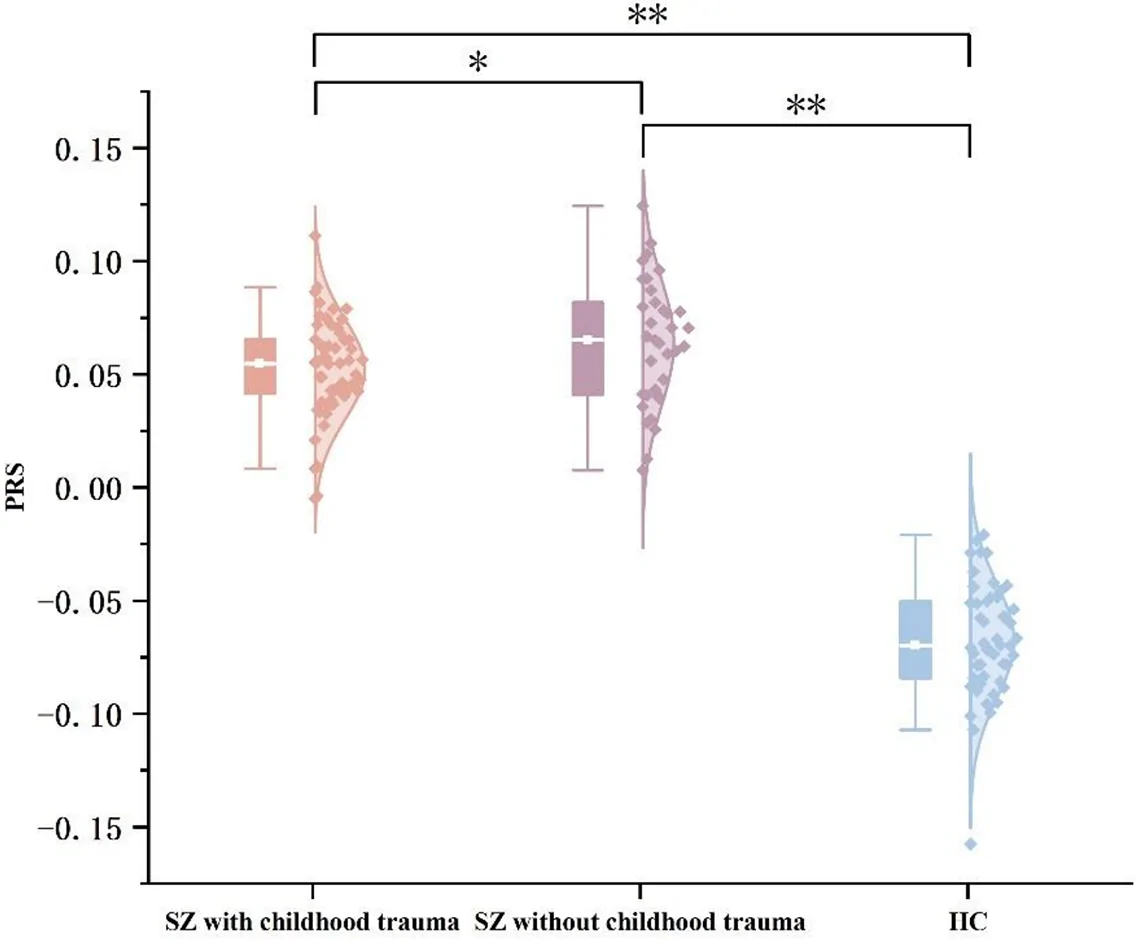
Differences of PRS among patients with schizophrenia with childhood trauma, schizophrenia without childhood trauma, and healthy control. *Note*: SZ, schizophrenia; HC, healthy control; PRS, polygenic risk score; ‘*’ indicates corrected *p* < 0.05, ‘**’ indicates corrected *p* < 0.001. Figure 2. long description.

As shown in Figure 2, PRS for schizophrenia was notably lower in patients with childhood trauma compared to patients without childhood trauma and healthy controls. The comparison of PRS between the schizophrenia groups with and without childhood trauma yielded a Cohen’s *d* of −0.479, 95% CI [−0.906, −0.049], indicating a moderate effect size. This indicates that people with a higher CTQ score do not have a higher polygenic liability for schizophrenia, contradicting the possibility of additive burden among individuals.

### Effect of childhood trauma and PRS on cognitive function

**Figure 3.**
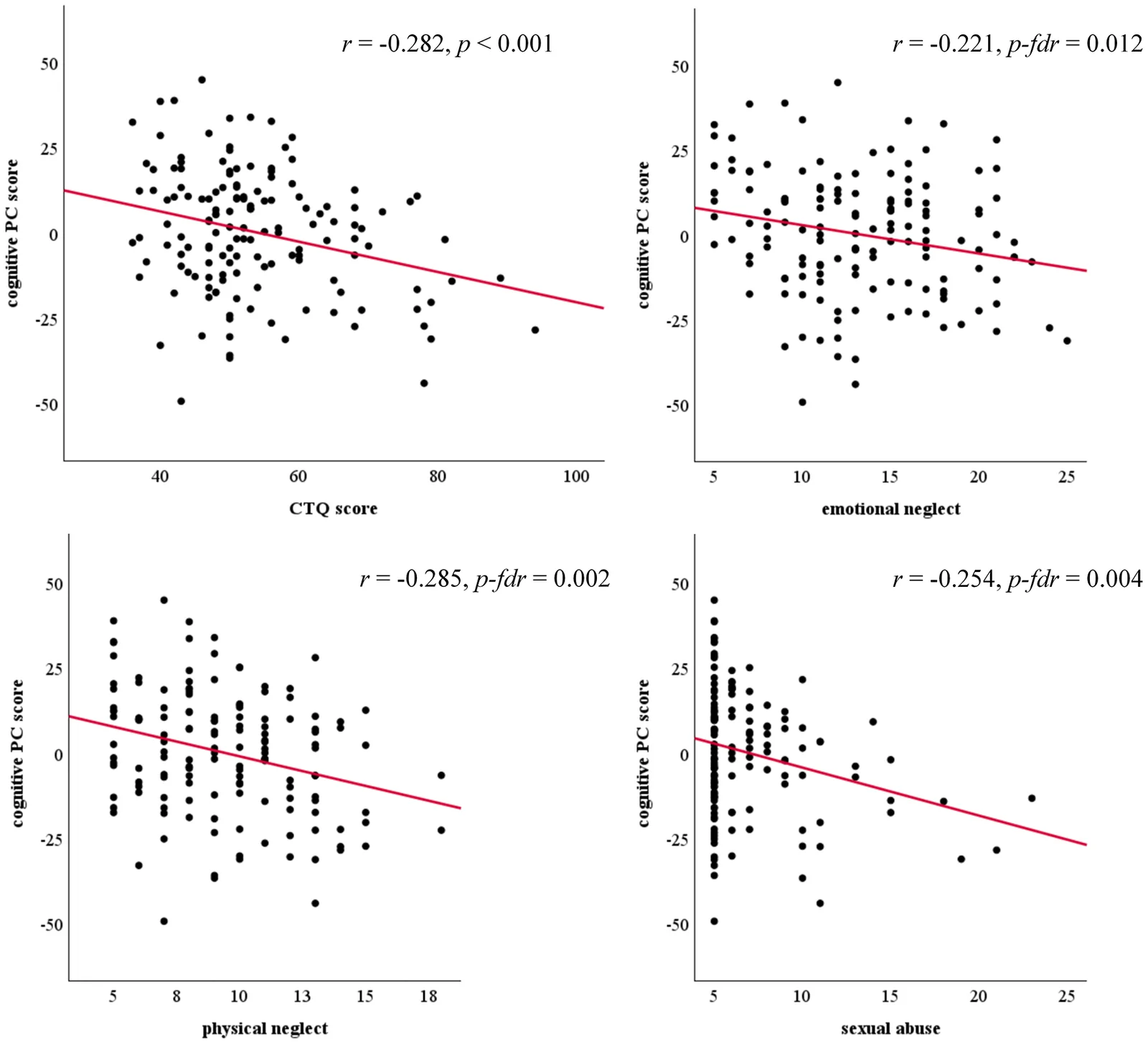
Correlation between the cognitive PC score and CTQ. The CTQ subscale correlations were FDR-corrected within the subscale analysis. The 95% confidence intervals for Pearson’s *r* were as follows: CTQ total score, [−0.425, −0.126]; emotional neglect, [−0.370, −0.061]; physical neglect, [−0.427, −0.129]; sexual abuse, [−0.399, −0.096]. *Note*: CTQ, Childhood Trauma Questionnaire; PC, principal component. Figure 3. long description.

In the entire schizophrenia sample, after excluding patients who did not complete the CTQ or cognitive assessments, a total of 147 patients remained. Within this sample, a significant negative correlation was found between the total score of CTQ and cognitive PC score (*r* = −0.282, *p* < 0.001, Figure 3). Specifically, the total CTQ score was significantly negatively correlated with the number of correct responses in Digit Symbol, Digit Span, Information, Arithmetic, and MWCST. Moreover, emotional neglect, physical neglect, and sexual abuse were all significantly negatively correlated with the cognitive PC score (Figure 3). However, no significant correlation was found between PRS and the cognitive PC score or any individual cognitive function measure.

### Effect of CTQ and PRS on sFC and dFC

**Figure 4.**
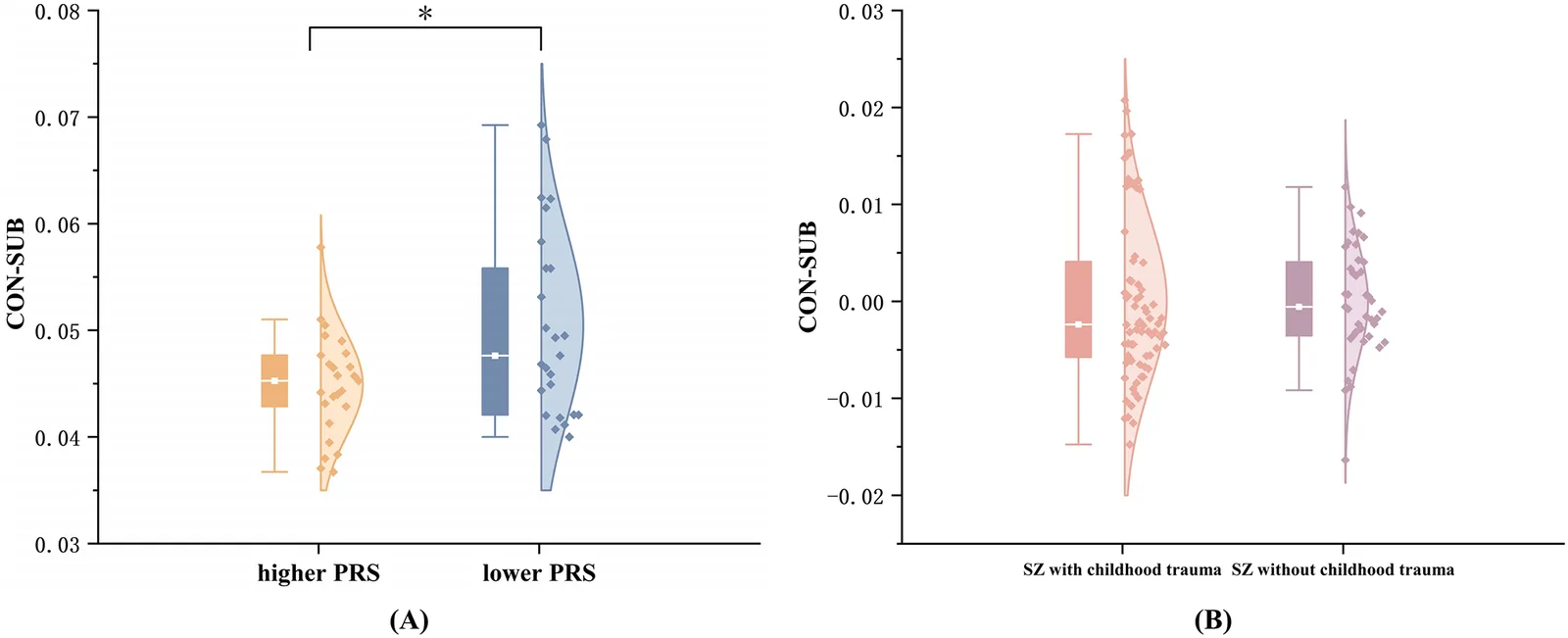
Group comparison of dynamic FC between CON and SUB. (A) Comparison between the top 25 and bottom 25 schizophrenia patients based on PRS. (B) Comparison between schizophrenia patients with childhood trauma and those without. *Note*: FC, functional connectivity; PRS, polygenic risk score; CON, cingulo-opercular network; SUB, subcortical network; ‘*’ indicates corrected *P* < 0.05. Figure 4. long description.

The comparison of edge-based static and dynamic FC among the three groups revealed significant differences (Supplementary Figure S1) and in patients versus healthy controls (Supplementary Figure S2). Between the two schizophrenia groups (with childhood trauma and without childhood trauma), no significant differences were found in edge-based sFC or dFC within SUB-related networks (Figure 4).

Among schizophrenia patients, the 25 individuals with the highest PRS were classified into one group, while the 25 individuals with the lowest PRS were classified into another. A significant difference in dFC between the CON and SUB networks was observed between the two groups (*F* = 7.949, *p* = 0.007, partial η^2^ = 0.153, 95% CI [0.013, 0.348]), with the higher-PRS group exhibiting lower dFC than the lower-PRS group (Figure 4).

### Additional analyses

Exploratory PRS × CTQ interaction analyses showed no significant interaction effects on the cognitive PC score or on any of the six subcortico-cortical dFC outcomes (Supplementary Table S2).

As a sensitivity analysis for the extreme-group PRS comparison, continuous PRS was not significantly associated with CON-SUB dFC after adjusting for age, sex, education, and mean FD, *F* (1, 119) = 0.242, *p* = 0.623.

In exploratory partial correlation analyses, CON-SUB dFC was not significantly associated with PANSS total, positive, negative, or general psychopathology scores after controlling for age, sex, education, and mean FD (Supplementary Table S3).

## Discussion

We expected a convergent effect of childhood trauma and polygenic risk of schizophrenia, whereby patients with higher CTQ would also have higher SczPRS, more cognitive dysfunction, and subcortical dysconnectivity. However, we noted higher SczPRS in patients *without* childhood trauma. Furthermore, while higher trauma related to overall cognitive dysfunction in line with a traumagenic neurodevelopmental model, higher PRS did not have the same effect on cognition. Subcortical connectivity was more affected in the presence of higher SczPRS than higher CTQ scores. These results can be interpreted based on the diathesis-stress model [79] and generate the hypothesis that trauma-related predisposition and common genetic liability show *partially distinct* associations with cognitive and neural phenotypes. Taken together, these findings suggest a tentative integrative model in which childhood trauma is more closely associated with cognitive impairment, whereas schizophrenia polygenic risk is more closely associated with cortico-subcortical dynamic connectivity. The absence of CTQ-related connectivity findings and PRS-related cognitive findings suggests that these risk factors may show domain-specific associations rather than uniform effects across cognitive and neural phenotypes.

We did not measure various types of environmental stress (e.g., academic failures, relationship breakdowns) that can produce dysregulation of the hypothalamic–pituitary–adrenal (HPA) axis and influence the risk for psychosis [80]. Such unobserved adversities may be more common in genetically predisposed individuals and have been linked in previous studies to dysregulation of the dopaminergic system, particularly in the associative striatum [81], which has been implicated in the development of positive symptoms [82]. Therefore, it is possible that environmental adversities not captured by CTQ may also be relevant to schizophrenia risk in some patients with lower PRS. Nevertheless, in our sample, higher PRS was not associated with greater exposure to childhood trauma. Together with the different associations of CTQ and PRS with cognition and subcortical dynamics, this pattern may support the possibility of partially distinct mechanistic pathways (bottom panel of Figure 1).

In the patients with schizophrenia, we observed a significant negative correlation between childhood trauma and cognitive function. Higher total CTQ scores were associated with lower scores on the cognitive PC and with poorer performance on several specific cognitive tasks, including processing speed, working memory, verbal comprehension, and executive function. These findings are consistent with previous studies suggesting that early-life adversity exerts a detrimental effect on cognitive outcomes in individuals with psychosis [83]. Among the CTQ subscales, emotional neglect, physical neglect, and sexual abuse showed significant associations with cognitive function, highlighting that those specific types of traumas may be especially harmful to neurocognitive development. This pattern reinforces the notion that the cognitive deficits observed in schizophrenia are not solely attributable to the disorder itself but may also be influenced by early environmental risk exposures. Our findings align with the neural diathesis-stress model [79] of schizophrenia, which emphasizes the long-term neurodevelopmental and cognitive impact of early-life adversity.

Interestingly, no significant association was found between PRS for schizophrenia and cognitive function, either at the level of the cognitive PC score or individual cognitive tests. This null finding should be interpreted in the context of the patient-only design and our limited sample size, where reduced variability in schizophrenia genetic liability and cognitive performance may make subtle PRS-cognition associations more difficult to detect. This result partly aligns with previous work suggesting that PRS, while predictive of diagnostic status, may have limited utility in explaining the heterogeneity of cognitive deficits among patients [84, 85]. A previous study [86] has shown that PRS for schizophrenia is related to cognitive deficits in schizophrenia even before the onset of psychosis (in high-risk cohorts); but other trait PRS also affect the genetic architecture of cognition in schizophrenia. A large cohort study [84] on schizophrenia demonstrated that the genetic variation in cognitive function in schizophrenia is related to factors that influence cognitive function in the general population and found no evidence of an association with liability to schizophrenia, major depressive disorder, or bipolar disorder. We lacked data on other trait PRS in this sample, limiting our observations to our specified hypotheses.

In the present study, we observed a significant difference in dFC between the CON and the SUB network when comparing schizophrenia patients with the highest and lowest PRS. Specifically, patients with lower PRS showed higher CON–SUB dFC than those with higher PRS. The CON, involving regions such as the anterior cingulate cortex and anterior prefrontal cortex, is crucial for cognitive control and performance monitoring [87], while the SUB network encompasses subcortical structures such as the striatum, thalamus, and amygdala, which are central to emotion regulation and reward processing [88].

Notably, these SUB regions, particularly the striatum, are key targets of dopaminergic projections and are consistently implicated in the neurobiology of schizophrenia [89]. The observed group difference in CON–SUB dFC may reflect altered functional integration within dopamine-sensitive circuits as a function of genetic risk.

This interpretation is supported by recent findings from Luykx [90], who demonstrated that a schizophrenia PRS enriched for dopamine-related genes predicted increased striatal dopamine synthesis and greater striatal activation during reward anticipation. Their results translate genetic risk for schizophrenia into dopamine-linked neurochemical and hemodynamic phenotypes, particularly within the striatum.

Taken together, our findings suggest that higher PRS may be associated with decreased functional flexibility between CON and SUB, potentially indicating less dynamic coordination in dopamine-sensitive networks. This provides further empirical support for a mechanistic pathway in which schizophrenia genetic risk may exert its effects through dopaminergic modulation of large-scale brain network dynamics.

A key strength of this study is the joint assessment of childhood trauma, schizophrenia polygenic risk, cognition, and resting-state dynamic connectivity within the same patient sample. Previous studies have examined the gene–environment interplay in schizophrenia [23], functional dysconnectivity models [11], or PRS-cognition associations [84] separately, whereas the present study extends this work by comparing environmental and genetic risk factors across both cognitive performance and dynamic cortico-subcortical connectivity phenotypes. Clinically, the findings suggest that childhood trauma history may help contextualize cognitive heterogeneity in schizophrenia and inform more individualized psychosocial and cognitive interventions.

It is important to acknowledge several limitations inherent in this study. First, the assessment of childhood trauma relied on the self-reported CTQ, which may be influenced by recall bias, current clinical state, and paranoid ideation, potentially leading to over- or underreporting of traumatic experiences. As the CTQ captures only selected dimensions of childhood maltreatment, future work could incorporate clinician-rated or interview-based assessments, supplemented when feasible by collateral developmental history, to provide a more comprehensive characterization of early-life adversity. Second, the cross-sectional design of the study prevents us from establishing causal relationships between PRS, childhood trauma, and their effects on cognitive functions and brain activity. Longitudinal studies are needed to better understand how these factors evolve over time. Third, the relatively small sample size may limit the statistical power and generalizability of the findings. Future research should aim to include larger sample sizes to replicate and confirm these results. Comparing the 25 patients with the highest PRS and the 25 patients with the lowest PRS provided an interpretable contrast in polygenic loading, but this dichotomization may be sensitive to sample size and effect-size inflation and requires replication in larger samples. Finally, the sample consisted of Chinese participants, and the generalizability of the PRS-related findings to other ethnic populations remains to be tested. We also did not systematically account for illness- and treatment-related factors, including duration of untreated illness, duration of illness, number of hospitalizations, specific antipsychotic type, and chlorpromazine-equivalent dose, which may influence cognition and functional connectivity. Future large-scale studies should collect more detailed illness-course and treatment information and combine CTQ with semi-structured interviews and collateral information where feasible. Additionally, this study focused on a limited range of cognitive functions and brain regions. Future research should expand the scope to explore how PRS and childhood trauma may influence other cognitive function and brain networks.

In conclusion, this study delineates the possibility of distinct roles of common genetic and trauma-related environmental factors in schizophrenia. Childhood trauma specifically associates with impairments in cognitive performance, whereas PRS is linked to alterations in dynamic functional connectivity within the subcortical/cingulo-opercular system. Clinically, these findings highlight the value of considering childhood trauma history when evaluating cognitive difficulties in patients with schizophrenia, which may help inform trauma-informed psychosocial and cognitive interventions. Additionally, the association between higher schizophrenia polygenic risk and altered subcortical connectivity generates a testable hypothesis that high vs. low-PRS patient groups may show differential sensitivity to dopamine-targeting antipsychotics; this needs to be investigated in future studies. Our findings suggest that genetic vulnerability and early-life adversity contribute to schizophrenia through dissociable pathways, underscoring the likelihood of a double hit in genetically predisposed individuals who experience trauma.

## Supporting information

10.1192/j.eurpsy.2026.12246.sm001 Huang et al. supplementary material Huang et al. supplementary material

## Supplementary material

The supplementary material for this article can be found at http://doi.org/10.1192/j.eurpsy.2026.12246.

## Long descriptions

### Figure 1. Long description

The diagram is divided into two horizontal panels by a blue line.

Top Panel (Convergence Model):

- On the left, two pentagonal boxes labeled Ch. Trauma and Polygenic risk point to a single white arrow.

- The arrow leads to a central node labeled Cognitive and Subcortical salience dysregulation, represented by a black and blue geometric network icon.

- A large red arrow points from this node to a dashed blue circle labeled Schizophrenia phenotype, containing three ovals: Phenotype 1, Phenotype 2, and Phenotype 3.

- To the right, three vertical columns show statistical expectations: Additive effect has a blue checkmark; Cognition C T Q | P R S shows two downward blue arrows; Subcortical dysconnectivity C T Q | P R S shows two downward blue arrows.

Bottom Panel (Separable Effects Model):

- On the left, Ch. Trauma points to Cognitive Dysfunction, and Polygenic risk points to Subcortical Salience Dysregulation.

- Both pathways lead into a large red arrow pointing to the same Schizophrenia phenotype circle.

- The statistical columns on the right show: Additive effect has a blue X; Cognition C T Q | P R S shows one downward arrow for C T Q and an X for P R S; Subcortical dysconnectivity C T Q | P R S shows an X for C T Q and one downward arrow for P R S.

Navigate back to Figure 1..

### Table 1. Long description

The table contains six rows of data across four main columns.

1. Age in years: S Z with trauma 18.63 plus or minus 2.59; S Z without trauma 18.65 plus or minus 3.04; H C 19.01 plus or minus 1.94. Group comparison F (2, 173) = 0.441, p = 0.644.

2. Education in years: S Z with trauma 11.51 plus or minus 2.23; S Z without trauma 11.67 plus or minus 1.95; H C 12.14 plus or minus 2.78. Group comparison F (2, 173) = 1.180, p = 0.310.

3. Sex (male/female): S Z with trauma 40/36; S Z without trauma 20/21; H C 34/25. Group comparison chi-squared (2) = 0.793, p = 0.673.

4. Mean F D in millimeters: S Z with trauma 0.074 plus or minus 0.034; S Z without trauma 0.077 plus or minus 0.038; H C 0.066 plus or minus 0.027. Group comparison F (2, 173) = 1.685, p = 0.188.

5. C T Q scores: S Z with trauma 58.37 plus or minus 10.45; S Z without trauma 44.05 plus or minus 4.41; H C 48.27 plus or minus 8.28. Group comparison F (2, 173) = 43.252, p < 0.001.

6. P A N S S scores: S Z with trauma 67.99 plus or minus 22.16; S Z without trauma 61.98 plus or minus 19.17; H C is not applicable. Group comparison t (115) = 1.465, p = 0.146.

Navigate back to Table 1..

### Figure 2. Long description

A vertical axis labeled P R S ranges from negative 0.15 to 0.15. The horizontal axis lists three groups from left to right.

1. S Z with childhood trauma (orange) shows a box plot centered around 0.05 with a violin plot showing a dense cluster of individual data points between 0.00 and 0.10.

2. S Z without childhood trauma (purple) shows a similar distribution with a median slightly above 0.05 and data points extending up to approximately 0.13.

3. H C (blue) shows a significantly lower distribution with a median near negative 0.07 and data points ranging from approximately 0.02 down to negative 0.15.

Statistical significance brackets at the top indicate the following. A single asterisk denotes corrected p less than 0.05 between the two S Z groups. Double asterisks denote corrected p less than 0.001 comparing H C to both S Z with childhood trauma and S Z without childhood trauma.

Navigate back to Figure 2..

### Figure 3. Long description

The figure consists of four scatter plots arranged in a two-by-two grid. All panels share a common y-axis labeled cognitive P C score, ranging from minus 50 to 50. Each panel includes a red linear regression line showing a downward trend and a cluster of black data points.

* Top-left panel: The x-axis is C T Q score, ranging from 40 to 100. The statistical annotation at the top right reads r equals minus 0.282, p less than 0.001.

* Top-right panel: The x-axis is emotional neglect, ranging from 5 to 25. The statistical annotation at the top right reads r equals minus 0.221, p-f d r equals 0.012.

* Bottom-left panel: The x-axis is physical neglect, ranging from 5 to 18. The statistical annotation at the top right reads r equals minus 0.285, p-f d r equals 0.002.

* Bottom-right panel: The x-axis is sexual abuse, ranging from 5 to 25. The statistical annotation at the top right reads r equals minus 0.254, p-f d r equals 0.004.

Navigate back to Figure 3..

### Figure 4. Long description

A two-panel figure labeled A and B. Both panels use a vertical y-axis labeled C O N minus S U B representing functional connectivity values.

Panel A: The y-axis ranges from 0.03 to 0.08. Two groups are compared on the x-axis: higher P R S (orange) and lower P R S (blue). Each group is represented by a box plot, a jittered scatter plot of individual data points, and a half-violin density plot. The lower P R S group shows higher connectivity values than the higher P R S group. A horizontal bracket with an asterisk above the two groups indicates a statistically significant difference.

Panel B: The y-axis ranges from minus 0.02 to 0.03. Two groups are compared on the x-axis: S Z with childhood trauma (red) and S Z without childhood trauma (purple). The data is presented using the same raincloud plot format as Panel A. The S Z without childhood trauma group shows slightly higher median connectivity values compared to the group with childhood trauma, though no significance bracket is present.

Navigate back to Figure 4..
