# Supplementary material for: Pathways to schizophrenia: Divergent effects of childhood trauma and polygenic risk on cognition and subcortical dynamics
Source: Eur Psychiatry. 2026 Jul 29;69(1):e80. doi: 10.1192/j.eurpsy.2026.12246 (PMC13735832; doi:10.1192/j.eurpsy.2026.12246)
Supplement: Supplementary file 1 [file S0924933826122469sup001.docx]

**S1. Detailed information on cognitive function assessment**

The assessment included several subtests from the Wechsler Adult Intelligence Scale (WAIS). Digit Symbol measured processing speed and visual-motor coordination by requiring participants to match symbols to corresponding digits within a set time limit. The Information subtest evaluated general knowledge and semantic memory through a series of factual questions. Arithmetic assessed quantitative reasoning and working memory by presenting participants with orally delivered math problems to solve. Digit Span tested working memory and attention by asking participants to repeat sequences of digits in both the same and reverse order. Higher scores on these subtests indicated better working memory capacity.

From the Wechsler Memory Scale-Revised (WMS-R), the Logical Memory subtest was used to assess verbal episodic memory. Participants were asked to recall a short narrative both immediately and after a delay, with higher scores reflecting better memory retention.

In addition to the WAIS and WMS-R subtests, other tasks were included to assess specific cognitive domains. The Verbal Fluency Task measured language fluency and executive function by asking participants to name as many animals as possible within 60 seconds. The Visual Patterns Test assessed visuospatial memory and reasoning, recording both the number of patterns accurately recalled and the most complex pattern successfully reproduced. Finally, the Modified Wisconsin Card Sorting Test (MWCST) was used to measure cognitive flexibility and executive function by tracking the number of correctly matched cards according to changing rules. This battery of tests provided a comprehensive measure of participants' cognitive functions across multiple domains.

**S2. Detailed information on MRI data acquisition.**

For each participant, the resting state functional MRI and T1-weighted structural imaging data were acquired from a 3.0 T Siemens scanner. The parameters of the scanning of rs-fMRI images were: slices = 32, matrix = 64 × 64, slice thickness = 5 mm, gap = 0 mm, gap = 0 mm, FOV = 240 × 240 mm², flip angle = 90°, echo time = 30 ms, repetition time =2000 ms, and total volumes = 216. And the paraments of the scanning of T1-weighted data were: slices = 176, matrix = 256 × 256, slice thickness = 1 mm, FOV = 256 × 256 mm², gap = 0 mm, echo time = 2.03 ms and repetition time =1900 ms.

**Figure S1. Comparisons on edge-based static and dynamic FCs among 3 groups**

Note: AUD, auditory network; CON, cinguloopercular network; DAN, dorsal attention network; DMN, default mode network; FPN, frontoparietal network; SAN, salience network; SMN, sensorimotor network; SUB, subcortical network; VAN, ventral attention network; VIS, visual network.

**Figure S2. Comparisons on edge-based dynamic FC among 3 groups**

Note: SZ, schizophrenia; HC, healthy control; AUD, auditory network; CON, cinguloopercular network; DAN, dorsal attention network; DMN, default mode network; FPN, frontoparietal network; SAN, salience network; SMN, sensorimotor network; SUB, subcortical network; VAN, ventral attention network; VIS, visual network; ‘*’ indicates corrected p < 0.05, ‘**’ indicates corrected p < 0.001.

**Table S1.** The cognitive characteristics of three groups

|  | SZ with childhood trauma, mean± SD | SZ without childhood trauma, mean± SD | HC, mean± SD | Group comparisons | |
| --- | --- | --- | --- | --- | --- |
| cognitive PC score | 0.36±16.34 | 7.58±17.81 | -1.42±17.01 | F (2, 156) = 3.48, p = 0.033 |  |
| Logical memory (Immediate) | 5.72±3.48 | 5.13±2.79 | 7.94±3.25 | F (2, 153) = 9.999, p < 0.001 |  |
| Logical memory (Delayed) | 3.36±2.79 | 3.74±2.29 | 5.88±3.09 | F (2, 149) = 12.369, p < 0.001 |  |
| Digit Symbol | 63.79±15.82 | 71.05±17.66 | 88.48±16.77 | F (2, 154) = 32.242, p < 0.001 |  |
| Information | 17.28±5.69 | 17.50±5.66 | 19.51±4.24 | F (2, 155) = 2.867, p = 0.060 |  |
| Digit Span (Forward) | 11.74±2.67 | 12.33±1.62 | 12.30±1.71 | F (2, 156) = 1.366, p = 0.258 |  |
| Digit Span (Backward) | 6.43±2.76 | 6.70±2.88 | 8.94±3.01 | F (2, 155) = 12.195, p < 0.001 |  |
| Verbal Fluency (Correct) | 17.93±5.60 | 18.53±4.76 | 22.71±5.27 | F (2, 152) = 12.451, p < 0.001 |  |
| Visual Patterns (Correct) | 18.13±5.16 | 18.40±4.16 | 21.82±6.39 | F (2, 155) = 7.688, p < 0.001 |  |
| Visual Patterns (Highest Completed Level) | 7.32±2.18 | 7.70±2.63 | 9.82±2.93 | F (2, 155) = 14.863, p < 0.001 |  |
| Arithmetic | 13.69±4.51 | 15.67±3.51 | 17.78±3.28 | F (2, 153) = 15.751, p < 0.001 |  |
| MWCST (Correct) | 31.65±8.18 | 33.49±6.68 | 38.59±6.35 | F (2, 145) = 12.960, p < 0.001 |  |

SZ, schizophrenia; HC, healthy control; PC, Principal Component; MWCST, Modified Wisconsin Card Sorting Test. F values are reported with degrees of freedom in the format F (between groups df, within groups df).

**Table S2.** Exploratory PRS × CTQ interaction analyses for cognitive and subcortico-cortical dFC outcomes

|  | PRS × CTQ interaction,  F(df1, df2) | p |
| --- | --- | --- |
| cognitive PC score | F(1,105) = 0.236 | 0.628 |
| SUB-DMN dFC | F(1,115) = 0.190 | 0.663 |
| SUB-FPN dFC | F(1,115) = 1.162 | 0.283 |
| SUB-CON dFC | F(1,115) = 0.292 | 0.590 |
| SUB-SAN dFC | F(1,115) = 1.293 | 0.258 |
| SUB-VAN dFC | F(1,115) = 0.411 | 0.523 |
| SUB-DAN dFC | F(1,115) = 0.274 | 0.602 |

PRS, Polygenic Risk Score; CTQ, Childhood Trauma Questionnaire; FC, functional connectivity; PC, Principal Component; SUB, subcortical network; DMN, default-mode network; FPN, frontoparietal network; CON, cingulo-opercular network; SAN, salience network; VAN, ventral attention network; DAN, dorsal attention network.

**Table S3.** Partial correlations between CON-SUB dFC and PANSS symptom scores

|  | partial r | p |
| --- | --- | --- |
| PANSS total score | -0.014 | 0.865 |
| PANSS negative score | 0.010 | 0.898 |
| PANSS positive score | -0.047 | 0.560 |
| PANSS general psychopathology score | 0.003 | 0.974 |

CON, cingulo-opercular network; SUB, subcortical network; PANSS, Positive and Negative Syndrome Scale; dFC, dynamic functional connectivity.
